# Knee function 30 years after ACL reconstruction: a case series of 60 patients

**DOI:** 10.1080/17453674.2021.1959709

**Published:** 2021-07-28

**Authors:** Thomas Söderman, Suzanne Werner, Marie-Louise Wretling, Mari Hänni, Christina Mikkelsen, Anders Sundin, Adel Shalabi

**Affiliations:** a Department of Radiology, Institution of Surgical Sciences, Uppsala University, Akademiska Hospital, Uppsala;;; b Department of Radiology, Karolinska University Hospital, Stockholm;;; c Department of Molecular Medicine and Surgery, Stockholm Sports Trauma Research Center, Karolinska Institutet, Stockholm, Sweden

## Abstract

Background and purpose — Until now, there have been no studies beyond 30 years after anterior cruciate ligament (ACL) reconstruction. We report knee function a mean 31 years after ACL reconstruction.

Patients and methods — This cohort comprised a case series of 60 patients with a mean follow-up of 31 years (28–33) after ACL reconstruction. Patients were evaluated with the International Knee Documentation Committee (IKDC) objective assessment, Knee injury Osteoarthritis Outcome Score (KOOS), Tegner Activity Scale, radiography, and MRI.

Results — 30 patients showed an intact ACL graft and 30 a ruptured or missing ACL graft. 40 patients had osteoarthritis in the tibiofemoral compartment and 24 patients in the patellofemoral compartment. Patients with intact ACL grafts scored higher than those with ruptured or missing ACL grafts when it comes to KOOS Sport/Rec. The Hodges Lehmann estimated median difference between groups was 15 (95% CI 0–35). The KOOS scores were lower in the group with ruptured or missing ACL grafts when compared with a healthy-knee reference group of males in terms of Pain, mean difference –8 (CI –15 to –1), Symptoms, mean difference –18 (CI –27 to –9), and Sport/Rec, mean difference –21 (CI –34 to –8). In the group with intact ACL grafts, the KOOS score was lower than a healthy-knee reference group of males in terms of Symptoms, mean difference –12 (CI –21 to –3). Scores for all subgroups of KOOS were higher in patients without osteoarthritis. The IKDC overall clinical assessment outcome was worse in patients with a ruptured or missing ACL graft. The Hodges Lehmann estimated median difference between groups was 1 (CI 0–1).

Interpretation — Patients with an intact ACL graft reported higher sports activity and recreation, as measured with KOOS, than patients with a ruptured or missing ACL graft. Patients with severe osteoarthritis reported lower sports activity and recreation, as measured with KOOS.

The anterior cruciate ligament (ACL) has a reported injury rate between 78 and 81/100,000 per individual and year (Nordenvall et al. 2012). In approximately half of the patients osteoarthritis (OA) is present 10–20 years after the ACL injury (Lohmander et al. 2007). It seems as if the development of OA does not differ whether patients have undergone ACL reconstruction or nonoperative treatment (Nordenvall et al. 2012, Smith et al. 2014, Lien-Iversen et al. 2019).

The development of OA in the injured joint is caused by intra-articular pathogenic processes initiated at the time of injury, combined with long-term changes in dynamic joint loading (Lohmander et al. 2007). Patients with ACL disruption sustain an acute chondral lesion that affects the overall cartilage homeostasis, resulting in a global degradation of the joint (Potter et al. 2012). There is increased anterior tibial subluxation in patients with failed ACL reconstructions compared with patients with acute ACL disruptions (Tanaka et al. 2013). Furthermore, abnormal tibiofemoral relationships alter the knee joint cartilage’s stress distributions, predisposing to degenerative changes (Andriacchi et al. 2004). Moreover, meniscal tears occur frequently in the setting of anterior cruciate ligament ACL ruptures, where medial meniscal tears are of most importance because the posterior horn of the medial meniscus is an essential secondary stabilizer against anterior tibial translation (Papageorgiou et al. 2001).

A study by Shelbourne et al. (2017) is one of a few studies beyond 20 years after ACL reconstruction reporting radiographic, physical, and patient-reported outcomes.

We report the outcome of ACL reconstruction, approximately 30 years after surgery, in terms of knee function evaluated with clinical assessments, radiological examinations, and patient self-reported outcomes. The hypothesis was that patients with ruptured or missing ACL grafts would report lower outcome scores as measured with KOOS than patients with intact ACL grafts. Furthermore, we hypothesized that patients with radiological OA would report lower outcome scores as measured with KOOS than those without OA.

## Patients and methods

This retrospective case series on clinical and radiological assessments was performed 28–33 years after ACL reconstruction.

### Patients

134 consecutive patients, who underwent ACL reconstruction at the Karolinska University Hospital between 1968 and 1973, were included. Primarily, all patients had been treated nonoperatively for their ACL rupture but were later referred to surgery because their knee joint instability prevented them from returning to their desired physical activities. Some of the patients showed previous injuries to their knee and entered the study diagnosed with medial collateral ligament injury, meniscal tears, and/or OA.

**Figure 1.**
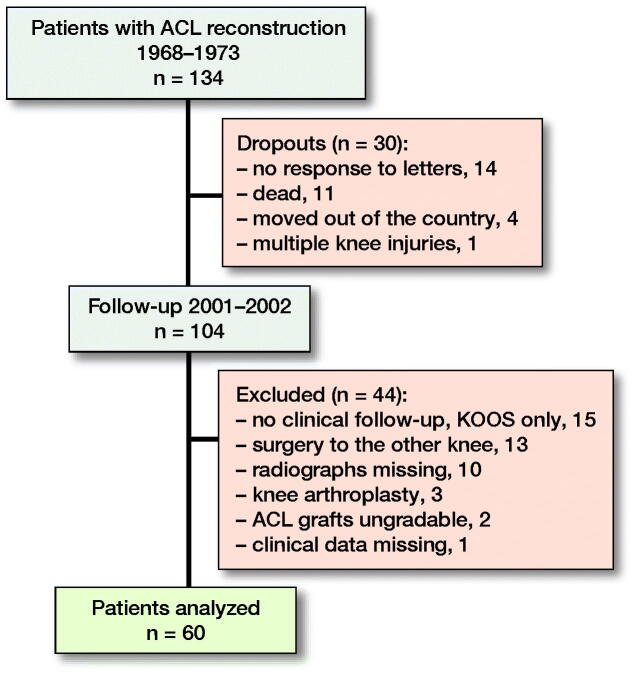
Flowchart of study patients.

In the first follow-up of this cohort in 1978, 87 patients were included, and the time from original injury to reconstructive surgery varied from 1 to 245 months (median 29 months; mean 41 months) (Johnson et al. 1984). At the present follow-up 2001/2002, 89 out of the original 134 patients were evaluated at the Karolinska University Hospital. 29 of these patients were excluded, and 60 patients (55 men) remained for the present assessment (Figure 1). The mean age was 27 years (SD = 6) at index surgery and 58 years (SD = 6) at follow-up. 35 patients had injured their right knee and 25 their left knee.

### Surgical technique

**Figure 2.**
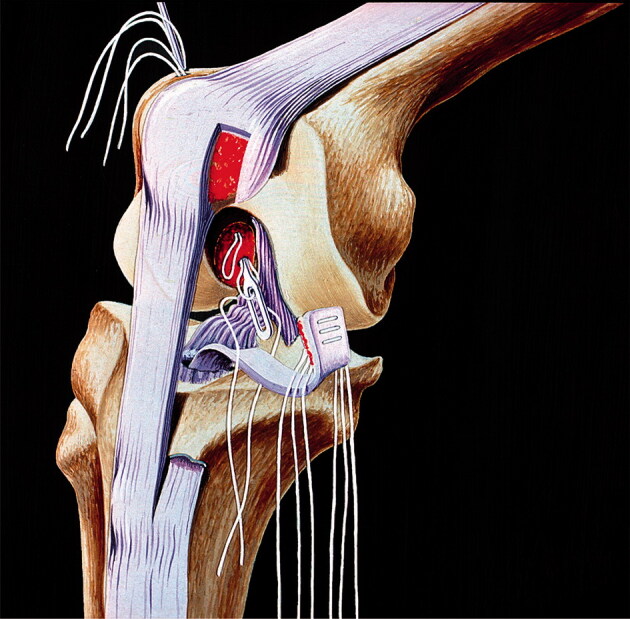
Schematic illustration of how the drill channel through the tibia was designed and how the tendon was drawn through it. Courtesy Ejnar Eriksson, MD.

The ACL reconstruction was performed according to a standardized method developed at the Karolinska University Hospital by Gillquist et al. in 1966 (Gillquist et al. 1971). They used the medial third of the patellar tendon, dissecting off two-thirds of the ligament that covered the patella, the patellar retinaculum. A hole was made from the tibial tubercle into the knee joint (close to the normal tibial attachment of the ACL), and the tendon flap was pulled into the knee joint. By a separate incision over the outside of the lateral condyle, two holes for sutures were drilled. A shallow hole at the normal posterior insertion of the ACL was also excavated. The sutures through the ligament were placed at varying sites on the ligament to facilitate stretching of the ligament flap when the sutures were tied one by one (Figure 2). Each bundle of sutures was pulled out using an extra thread, and the operation was then completed.

In those cases where associated surgery was performed simultaneously with index surgery, meniscus resection and repair of the medial collateral ligament were performed (Gillquist et al. 1971).

### Postoperative treatment

**Figure 3.**
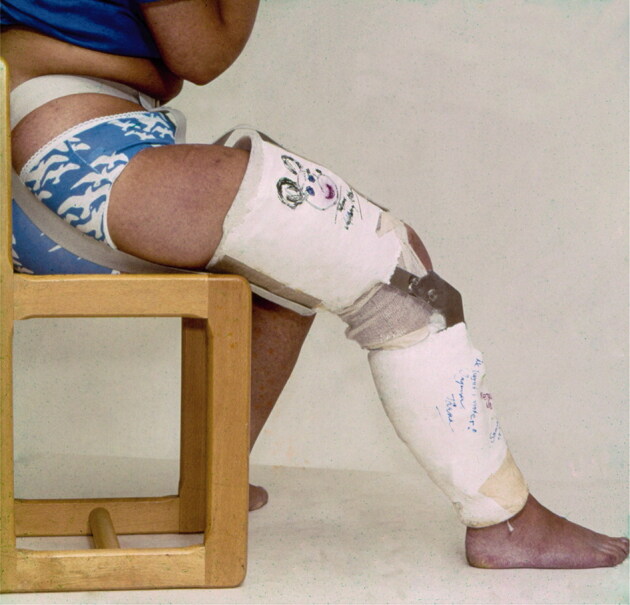
4 to 5 weeks postoperatively, a partly mobile cast brace was applied that allowed 40° of flexion, from 20° to 60°. Courtesy Ejnar Eriksson, MD.

A posterior plaster sprint was used for 1 week. The splint was then changed for a partially mobile cast-brace, allowing the knee to flex 20°–60°, and the patients were allowed to put weight on the operated leg (Figure 3). The cast-brace was used for 4 to 5 weeks postoperatively. Physiotherapy started immediately after surgery and continued for 6 continuous weeks while the cast-brace was still in place. The goal of rehabilitation was to improve the range of motion of the knee joint and increase thigh muscle strength, including balance and coordination exercises (Eriksson 1976).

### Clinical outcome measures

Objective evaluations were performed using the 2000 International Knee Documentation Committee (IKDC) objective form (Hefti et al. 1993). Clinical evaluations were conducted by an orthopedic surgeon with extensive experience in performing objective measures of the knee joint. Subjective evaluations utilized the KOOS (Roos et al. 1998) and Tegner Activity Scale (Tegner and Lysholm 1985). The Tegner Activity Scale was used in a somewhat modified version due to the inclusion of certain new sports such as floorball and martial arts that had become popular since 1985 when the Tegner Activity Scale was published. The KOOS subscale Quality of Life will be reported elsewhere.

### Radiological examinations

At the present follow-up 2001/2002, all patients underwent radiographic and MRI examinations of the index knee. Weight-bearing radiographs in anteroposterior, lateral, and a skyline view of the patellofemoral compartment were acquired (Ahlbäck and Rydberg 1980). MRI was performed on a low field, 0.2 T scanner (Arthroscan; Esaote Group, Genoa, Italy), to obtain sagittal (T2 STIR 5 mm, T2 5 mm, T1 5 mm, T1 4 mm, and T1 3 mm) and coronal (T2 STIR 5 mm and T1 5 mm) images. The radiological examinations were read in consensus by 2 senior consultants in musculoskeletal radiology.

The radiological examinations were assessed to determine OA degree using the Kellgren–Lawrence classification. Radiographic tibiofemoral and patellofemoral OA was defined as Kellgren–Lawrence ≥ 2 in any of the compartments.

MRI was used to assess the menisci and the structural integrity of the ACL graft. The menisci were classified as 1 (normal), 2 (small/defect), 3 (rupture), or 4 (missing). The ACL graft was graded as intact, ruptured, missing, or impossible to evaluate due to artifacts.

### Statistics

Data was analyzed using GraphPad Prism version 7.00 for Macintosh (GraphPad Software, LaJolla, CA, USA; www.graphpad.com). Descriptive statistics such as frequency, mean (SD), median (range) were employed. A Mann–Whitney U-test was used for group comparisons in terms of intact and ruptured or missing ACL grafts, respectively. Mann–Whitney was also used for comparison between groups with OA and without OA. To define the magnitude of effects, an estimate of the median difference between groups, together with the 95% confidence interval (CI), was calculated with the use of the Hodges–Lehmann approach.

Based on KOOS, Student’s t-test was used comparing knee function of the study cohort with a healthy Swedish population. To define the magnitude of effects, the mean difference between groups, together with the 95% confidence interval, was calculated. Spearman’s test was used for correlation analysis. Statistical significance was set at a p-value of < 0.05.

3 patients had missing data in single variables due to incomplete filling out of forms (n = 1) and clinical examination results not reported (n = 2). 4 patients had missing data in terms of the Tegner Activity Scale. The existing values were used in the statistical analysis. No mathematical correction was made for multiple comparisons.

### Ethics, funding, and potential conflicts of interest

Ethical approval was obtained from the Regional Ethics Committee in Stockholm, Sweden (Dnr 2016/2108-31/4). All patients gave informed consent to participate. No funding has been obtained for this study. The authors declare no potential conflicts of interest.

## Results

### International Knee Documentation Committee

The results of IKDC were available for all 60 patients (Table 1). The IKDC overall clinical assessment outcome was worse in patients with a ruptured or missing ACL graft. The Hodges Lehmann estimated median difference between groups was 1 (CI 0–1).

**Table 1.** Clinical outcome of knee function according to IKDC

Factor	Normal	Nearly normal	Abnormal	Severely abnormal
Effusion, n = 59				
Intact ACL	22	6	0	1
Ruptured or missing ACL	19	10	0	1
Passive motion deficit extension, n = 59				
Intact ACL	11	13	4	2
Ruptured or missing ACL	12	7	7	3
Passive motion deficit flexion, n = 60				
Intact ACL	10	15	5	0
Ruptured or missing ACL	6	13	10	1
Ligament examination,^a^ n = 60				
Intact ACL	22	1	7	0
Ruptured or missing ACL	9	2	19	0
IKDC overall, n = 60				
Intact ACL	4	14	10	2
Ruptured or missing ACL	1	4	18	7

a Lachman manual max, 25° flexion

There was no statistical group difference regarding extension, flexion, or effusion. Anterior knee laxity based on the Lachman test was higher in patients with ruptured or missing ACL grafts than those with intact ACL grafts. The Hodges Lehmann estimated median difference between groups was 1 (CI 0–2).

### Knee injury Osteoarthritis Outcome Score

All 60 patients completed the KOOS (Table 2). Patients with intact ACL grafts scored higher than those with ruptured or missing ACL grafts when it comes to KOOS Sport/Rec. The Hodges Lehmann estimated median difference between groups was 15 (CI 0–35). There was no statistical group difference in terms of Pain, Symptoms, or ADL.

**Table 2.** KOOS scores for the groups with intact and ruptured or missing ACL grafts. Values are mean (SD)

KOOS subscales	Intact ACL (n = 30)	Ruptured^a^ (n = 30)	Reference group^b^	p-value^c^
Pain	86 (18)	80 (16)	88 (17)	0.06
Symptoms	76 (22)	70 (22)	88 (17)	0.2
ADL	92 (13)	87.5 (14)	86 (19)	0.2
Sport/Rec	68 (24)	52 (32)	73 (30)	0.04

a or missing ACL

b Reference population (n = 88 except for Sport/Rec with 1 missing value) of 55- to 74-year-old men added for comparison from Paradowski et al. 2006.

c Intact ACL graft versus ruptured or missing ACL graft.

The KOOS scores were lower in the group with ruptured or missing ACL grafts when compared with a healthy-knee reference group of males (Paradowski et al. 2006) in terms of Pain, mean difference –8 (CI –15 to –1), Symptoms, mean difference –18 (CI –27 to –9), and Sport/Rec, mean difference –21 (CI –34 to –8). The KOOS scores were lower in the group with intact ACL grafts than a healthy-knee reference group of males (Paradowski et al. 2006) in terms of Symptoms, mean difference –12 (CI –21 to –3). On the other subscales of KOOS, there were no statistical differences.

When the patients were divided into those with OA (n = 40) and without OA (n = 20), in the tibiofemoral compartment, patients without OA scored higher in terms of Pain, median difference 8 (CI 3–17), Symptoms, median difference 17 (CI 7–29), ADL median difference 4 (CI 0–9), and Sport/Rec, median difference 20 (CI 5–35). When the patients were divided into those with OA (n = 24) and without OA (n = 36) in the patellofemoral compartment, Pain, median difference 6 (CI 0–19), Symptoms, median difference 14 (CI 4–29), ADL, median difference 4 (CI 0–12), and Sport/Rec, median difference 20 (CI 5–35) were higher in the group without OA.

A weak association was found between Sport/Rec and OA in the medial tibiofemoral compartment (r = –0.25, p = 0.05), lateral tibiofemoral compartment (r = –0.26, p = 0.04), and the patellofemoral compartment (r = –0.35, p = 0.007).

### Tegner Activity Scale

56 patients completed the Tegner Activity Scale, while 4 did not (Table 3). The median activity level was 4, ranging from 2 to 7 for patients with intact ACL graft and 3 to 7 for patients with a ruptured or missing ACL graft. 11 of the patients with intact ACL graft and 14 of those with ruptured or missing ACL graft reported an activity level of recreational sports or higher (≥ level 5).

**Table 3.** Tegner Activity Scale for patients with intact and ruptured or missing ACL grafts

Activity level	Intact ACL graft (n = 27)	Ruptured or missing ACL graft (n = 29)
9–10	0	0
7–8	4	5
5–6	7	9
3–4	13	15
1–2	3	0
0	0	0

### Radiographic assessment

OA changes were assessed in the medial tibiofemoral compartment, the lateral tibiofemoral compartment, and the patellofemoral compartment (Table 4).

**Table 4.** Distribution of osteoarthritis for the groups with intact and ruptured or missing ACL grafts using the Kellgren–Lawrence classification

	Intact ACL graft (n = 30)				Ruptured or missing ACL graft (n = 30)			
K–L grade:	0 and 1	2	3	4	0 and 1	2	3	4
Patellofemoral	21	4	5	0	15	11	3	1
Medial tibiofemoral	20	3	7	0	7	8	8	7
Lateral tibiofemoral	22	4	3	1	19	6	1	4

40 patients had OA in the tibiofemoral compartment and 24 patients in the patellofemoral compartment. In patients with intact ACL grafts, 16 had OA in the tibiofemoral compartment and 9 in the patellofemoral compartment. In patients with ruptured or missing ACL grafts, 23 had OA in the tibiofemoral compartment and 15 in the patellofemoral compartment.

### MRI assessment

Of the 60 patients, 30 had intact ACL grafts, and in 30 patients the ACL graft was either ruptured or missing. Before or in connection with the index surgery, 15 out of 60 patients had been treated with meniscus resection of the medial meniscus and 4 of the lateral meniscus (Table 5).

**Table 5.** Distribution of meniscal injuries for the groups with intact and ruptured or missing ACL grafts

Meniscal injury grade	Intact ACL graft (n = 30)		Ruptured or missing ACL graft (n = 30)	
	Medial meniscus	Lateral meniscus	Medial meniscus	Lateral meniscus
1	9	17	2	14
2	9	2	19	3
3	5	9	1	11
4	7	2	8	2

## Discussion

Our main finding a mean 31 years after ACL reconstruction was that patients with an intact ACL graft reported higher sports activity and recreation, as measured with KOOS, than patients with a ruptured or missing ACL graft. Remarkably low graft survival and that patients with ruptured ACL grafts showed substantially more OA of the medial tibiofemoral compartment than in those with an intact graft has been reported previously (Söderman et al. 2019).

In a recently published 20-year follow-up study (van Yperen et al. 2018), similar KOOS results were reported for operative and nonoperative treatment of ACL ruptures. However, the clinical outcome of that study assessed with IKDC was better for surgical treatment. In our study, with exclusively surgical patients, higher KOOS Sport/Rec scores and better IKDC values were found for patients with intact ACL grafts than those with ruptured or missing ACL grafts, which may point to the importance of an intact ACL graft. This is supported by Granan et al. (2015), who reported an association between impaired knee function measured with KOOS and a prospective ACL graft failure. Furthermore, the functional results assessed with IKDC are supported by Kessler et al. (2008), who showed a clear clinical outcome advantage in favor of ACL reconstruction compared with nonoperative treatment. It must be considered that the IKDC total is determined by the worst individual parameter, which in our study was the ligament test. A large number of the patients in the study by Kessler et al. with evident objective anteroposterior instability (IKDC B, C, D) were also almost symptom-free and physically active at a high level.

When interpreting patient-reported outcomes, it is essential to do so in a clinically meaningful manner. The measurement of the patient’s acceptable symptom state (PASS) is an accepted way of doing that (Svantesson et al. 2020). Half of the patients in the group with intact ACL graft and one-third of the patients with ruptured ACL grafts scored KOOS Sport/Rec as 75 or more, which constitutes a previously identified threshold for the patient acceptable symptom state 1 to 6 years after ACL reconstruction (Muller et al. 2016). The remainder of our patients scored below 75. This is in line with a previous long-term follow-up mean 16 years after ACL reconstruction (Hamrin Senorski et al. 2019), where half of the patients had a PASS value above the cutoff value.

Interestingly, in patients with intact ACL grafts, the proportion of patients with PASS value above the cutoff value was nearly the same as in the study by Hamrin et al. (2019), even though the follow-up time was significantly longer in our study and the mean age was higher. The reason for this is unclear, but it could partly be explained by the fact that the cutoff value for PASS in our study was KOOS but IKDC in the study by Hamrin et al. Moreover, the PASS cutoff value could possibly vary with age due to different activity levels in different ages (Briggs et al. 2009).

It has been suggested that an 8–10-point change in KOOS constitutes a clinically relevant difference (Roos and Lohmander 2003). In the subscales Symptoms and Sport/Rec in the group with ruptured or missing ACL graft, the mean KOOS scores were more than 10 points lower than in the male reference group of KOOS (Paradowski et al. 2006). Furthermore, the difference between KOOS scores for healthy controls and for patients with intact ACL grafts was less than 10 points in all KOOS subgroups besides Symptoms. This further emphasizes the importance of an intact ACL graft.

Patients with radiologic OA reported lower KOOS scores than those without OA. This is in line with previous findings of associations between severe OA and KOOS scores in terms of Pain, Symptoms, ADL, Sport/Rec, and QOL after ACL reconstruction (Oiestad et al. 2011).

It has been shown that loss of knee extension and/or loss of flexion increases the odds of developing moderate to severe OA (Shelbourne et al. 2017). This is in line with our results, where 36 of 60 patients had abnormal knee extension, and 40 of 60 had OA in the tibiofemoral compartment.

Patients with intact and ruptured or missing ACL grafts showed similar median Tegner activity levels (4), the reasons for which are somewhat unclear. Taken together, the majority of the patients had an activity level between 3 and 6, corresponding to activities during daily living or recreational sports, and the average age at follow-up was 56 years, which may imply that age is essential or even determines activity level. This is consistent with a previous study, which showed that healthy persons’ Tegner activity level was inversely correlated with age (Briggs et al. 2009).

The following study limitations should be mentioned. These are the low rate—60/134—of follow-up, the heterogeneous cohort, and that the clinical evaluations were performed almost 20 years ago with the risk of lost data and the development of evaluation methods. Furthermore, some of the patients had previous injuries to their knee and entered the study diagnosed with collateral ligament injury, meniscal tears, and/or OA. Another limitation is the lack of a control group; all our patients had had surgery. A further limitation is that MRI was performed with a low field strength magnet (0.2 T). The strength of the study is that it presents data from a long-term follow-up after ACL reconstruction, mean 31 years, which to our knowledge is unique. Furthermore, the clinical assessments were unbiased to some extent because they were done by an orthopedic surgeon from another institution who was not involved in the ACL reconstruction.

The interpretation of our results cannot without reservation be generalized and transformed to today’s population of ACL-reconstructed knees, as surgical techniques have been developed and improved continuously since the beginning of this study. Current surgery is performed arthroscopically and the graft is fully resected instead of leaving the insertion of the tendon in the tibial tuberosity intact. Anatomical ACL reconstructions are increasingly performed. However, it is also noteworthy that over time different grafts are being used, and it cannot be excluded that this might lead to a different result. In addition, the studied population is a group where nonoperative treatment was not sufficient to regain knee joint stability and desired knee function. The time between injury and surgery might have been longer than in current clinical practice. Therefore, it is not unlikely that future long-term outcomes after surgery applying modern techniques will result in a better clinical outcome.

In conclusion, a mean 31 years after ACL reconstruction, patients with an intact ACL graft reported higher sports activity and recreation than those with a ruptured or missing ACL graft, and patients with severe OA reported lower sports activity and recreation (KOOS).
